# An Anti-Checkpoint Activity for Rif1

**DOI:** 10.1371/journal.pgen.1002421

**Published:** 2011-12-15

**Authors:** Yaniv Harari, Linda Rubinstein, Martin Kupiec

**Affiliations:** Department of Molecular Microbiology and Biotechnology, Tel Aviv University, Ramat Aviv, Israel; Fred Hutchinson Cancer Research Center, United States of America

Chromosomal double-strand breaks (DSBs) are among the most severe lesions a cell has to deal with: if left unrepaired, they may lead to cell death or cancer. Thus, efficient mechanisms have evolved that respond to the presence of DSBs. These are collectively called the “DNA damage response” (DDR), or the “DNA damage checkpoint”. As a result of intensive studies by many research groups in several model organisms, the basic mechanisms that respond to DNA damage have been delineated: following the formation of DSBs, the broken ends are resected, exposing single-stranded DNA (ssDNA) which gets covered by Replication Protein A (RPA), eliciting cell cycle arrest through a complex cascade of protein recruitment and phosphorylation in which several kinases take part (reviewed in [1]). The ends of linear eukaryotic chromosomes, called telomeres, resemble DSBs; however, they do not normally elicit the checkpoint: the DNA ends are somehow “hidden” from the checkpoint-activating mechanisms. This is a very important feature, as it prevents continuous cell cycle arrests or inappropriate (and undesirable) repair of the natural chromosome ends. However, the precise mechanism(s) by which telomeres avoid checkpoint activation have remained elusive. In the accompanying paper, Xue et al. [2] identify *Saccharomyces cerevisiae* Rif1 as an important telomeric factor with an anti-checkpoint role.

Yeast telomeres maintain their integrity by the action of three different protein complexes: the CST (Cdc13-Stn1-Ten1) complex, which resembles RPA and binds to the telomeric G-rich single-stranded 3’ end; the Yku70/80 heterodimer, which blocks single-stranded DNA formation specifically in G1 [3]; and the Rap1 protein, which binds the TG-rich telomeric dsDNA and recruits two additional proteins, Rif1 and Rif2, via its C-terminus [4]. The Rif1 and Rif2 proteins seem to have important, yet different, roles in determining the integrity and length of telomeres [4]–[6]. Xue and co-workers [2] have studied the recruitment of several proteins to the telomeres in a strain carrying the temperature-sensitive *cdc13-1* allele. In such strains, upon transfer of the cells to the restrictive temperature (e.g., 36°C) telomeres become uncapped and DNA resecting factors such as Sgs1 and Exo1 are recruited, generating ssDNA [7]. The authors followed the recruitment of the various factors, as well as the binding of checkpoint proteins, by chromatin immuno-precipitation (ChIP) at telomeric, subtelomeric, and unrelated sequences after transfer of the cells to the restrictive temperature.

As expected, once resection by Sgs1 and Exo1 started, the amount of Rap1 bound to the telomeric sequences diminished (as Rap1 binds dsDNA); however, surprisingly, Rif1 accumulated with the same pattern as that of the DNA processing enzymes. This was true even in strains in which the C-terminus of Rif1 (thought to be essential for its recruitment) was deleted. Thus, Rif1 can associate to resected telomeres independently of Rap1.

**Figure 1:**
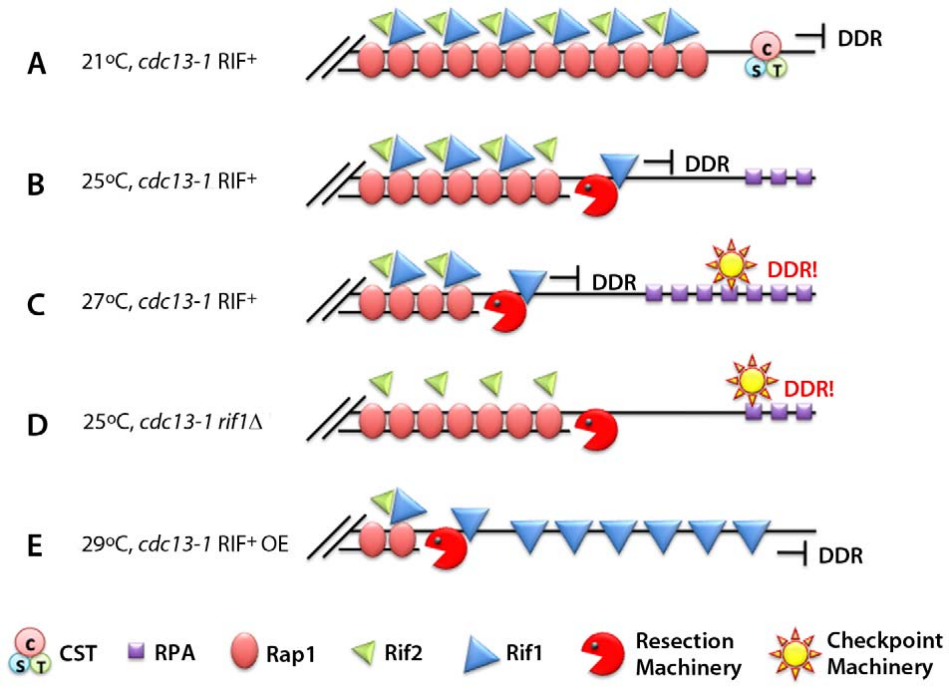
Rif1 works as an anti-checkpoint protein. (A) At 21°C the CST complex in a *cdc13-1 RIF1^+^* strain is still functional and “caps” the telomeres, preventing the DNA damage response (DDR). (B) At 25°C the CST is not entirely functional. The resection machinery (Sgs1, Exo1, etc.) creates ssDNA. The presence of Rif1 prevents DDR activation. (C) At 27°C the CST becomes non-functional, and the amount of Rif1 available cannot prevent binding of RPA and additional checkpoint proteins. (D) In the absence of Rif1, the checkpoint is elicited even at 25°C. (E) Over-expressing Rif1 allows the cells to grow at 29°C without eliciting the DDR.

The presence of Rif1 had a negative effect on the recruitment of the checkpoint sensors RPA, Ddc2^ATRIP^, Ddc1^RAD9^, and Rad9^53BP1^: a much higher recruitment of these proteins was seen in strains lacking Rif1 than in the wild type. Moreover, with time after temperature shift, the negative effect of Rif1 was stronger at proximal sites than at the subtelomeric sequences, suggesting that the Rif1 protein itself moves; these effects were not caused by increased ssDNA levels or by changes in the dynamics of resection. Thus, it appears that Rif1 travels with the resection machinery at telomeres, preventing the local activation of the checkpoint by interfering with the recruitment of RPA and checkpoint sensors (Figure 1). Rif1 seems to act by de-sensitizing cells to the presence of ssDNA: whereas *cdc13-1 RIF1+*/*RIF1-CΔ* cells respond to the presence of ssDNA when its level reaches 6%–10% (at 27°C) but not at low ssDNA levels (e.g., at 25°C), *cdc13-1 rif1Δ* cells already arrest in the cell cycle in the presence of only 2% ssDNA (at 25°C).

If Rif1 sets the threshold for the DDR, then overexpression of the protein might elevate the threshold: indeed, *cdc13-1* cells overexpressing Rif1 were able to grow at 29°C, an effect similar to the one obtained by deleting checkpoint components such as *RAD24^RAD17^* and *RAD17^RAD9^* [8]. Thus, Rif1 over-expression has the same effect as a checkpoint knockout, abrogating cell cycle arrest. Moreover, increasing expression of Rif1 in *cdc13-1* cells already arrested at the restrictive temperature allowed them to exit the cell cycle arrest, demonstrating that Rif1 can out-compete the checkpoint proteins already present at the telomeres and extinguish an ongoing checkpoint response. Interestingly, this effect was telomere specific, as no anti-checkpoint effect could be seen associated with non-telomeric-induced DSBs.

Some time ago Weinert and colleagues [9] showed that the presence of a telomeric tract close to an artificial DSB gradually turned off the DDR elicited by the DSB. The molecular nature of this anti-checkpoint effect was not clear at the time, but the Rif1 protein seems to fit all the requirements for such an anti-checkpoint factor: it is specific for telomeres, acts in *cis*, and does not affect the resection or the repair of the broken ends.

The identification of Rif1 as an anti-checkpoint factor is a huge step forward; however, many questions remain: If Rif1 activity is independent of Rap1, what is the mechanism of its recruitment? Unlike its vertebrate ortholog, the yeast Rif1 lacks a C-terminal DNA-binding domain [10]. Does yeast Rif1 require an additional factor for binding? Does it move with the DNA-resection machinery by being somehow linked to it? Interestingly, the vertebrate Rif1 protein was shown to interact with DNA and with the BLM protein (the ortholog of yeast’s Sgs1) [10]. An intriguing hypothesis is that Rif1 may be bound to Rap1 at normal telomeres; when telomeres become uncapped, the resection machinery may advance along the chromosome, dislodging Rap1 and concomitantly recruiting Rif1. What then is the role of Rif2? Genetic analysis has shown that its role is independent of Rif1 in determining telomere length [4]. Finally, what is the mechanism by which Rif1 can turn off an ongoing checkpoint response? An attractive idea proposed by Xue et al. [2] is that Rif1 may help recruit phosphatases to de-phosphorylate the central checkpoint kinases.

Interestingly, mammalian Rif1 was thought to function differently from yeast Rif1, as it can be found at non-telomeric locations and does not co-localize with Rap1 at normal telomeres [11]. The data presented here, however, suggest that Rif1 activity in yeast is independent of Rap1 and that yeast and mammalian proteins may share more features than originally thought. Remarkably, Rif1 expression is elevated in human breast tumors, and its expression status is also positively correlated with differentiation degrees of invasive ductal carcinoma of the breast [12]. If the anti-checkpoint role of Rif1 is conserved in mammalian cells, the increased levels of Rif1 may artificially increase the threshold for ssDNA recognition, allowing cells to continue their proliferation in the presence of unrepaired DNA damage without eliciting the DDR.
